# Sparse Nanocrystals Enable Ultra‐Low Coercivity and Remarkable Mechanical Robustness in High‐Entropy Amorphous Alloy

**DOI:** 10.1002/advs.202503546

**Published:** 2025-07-12

**Authors:** Lichen Liu, Liliang Shao, Yan Ma, Zhilin Wen, Jing Zhou, Yuqiang Yan, Weiming Yang, Haibo Ke, Weihua Wang

**Affiliations:** ^1^ School of Mechanics and Civil Engineering China University of Mining and Technology Xuzhou 221116 China; ^2^ Songshan Lake Materials Laboratory Dongguan 523808 China; ^3^ College of Materials Science and Engineering Hohai University Changzhou 213200 China; ^4^ Guangdong Key Laboratory for Advanced Metallic Materials Processing South China University of Technology Guangzhou 510640 China; ^5^ Institute of Physics Chinese Academy of Science Beijing 100190 China

**Keywords:** amorphous‐nanocrystalline transitional structure, coercivity, ferromagnetic high‐entropy alloys, order modulation, toughness

## Abstract

Ferromagnetic high‐entropy alloys (HEAs) are known for their excellent mechanical properties, which are attributed to their abundant ordered structures. However, they often exhibit compromised soft magnetic properties, which restrict their applications in modern electronics. In this study, an order‐modulation strategy is introduced to overcome this limitation by constructing an amorphous‐nanocrystalline transitional structure in a ferromagnetic HEA system. Subsequently, FeCoNiAlTaSiB high‐entropy sparse nanocrystals alloys are developed that possess fine nanocrystals sparsely dispersed in an amorphous matrix. This allows the resultant alloys to combine an ultra‐low coercivity (0.3 A m^−1^) with remarkable mechanical toughness, achieving a synergistic enhancement of the mechanical robustness and soft magnetic properties. This remarkable mechanical‐magnetic synergy is attributed to the presence of numerous crystal‐like orders (<2 nm), regular magnetic‐domain structures, and minimized magnetic anisotropy. Moreover, the proposed order‐modulation strategy successfully extend structural control across the entire order space, from amorphous to crystalline, providing a new paradigm for designing advanced soft magnetic materials with balanced mechanical and magnetic properties.

## Graphical abstract

An order modulation strategy is introduced to overcome trade‐off between mechanical and magnetic properties by constructing an amorphous‐nanocrystalline transitional structure in a ferromagnetic HEA system. This enable the resultant alloys to combine an ultra‐low coercivity of 0.3 A m^−1^ with a remarkable mechanical toughness, thereby achieving a synergistic enhancement in terms of the mechanical robustness and soft magnetic properties.

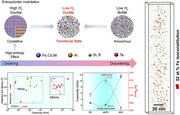

## 1 Introduction

Over the past few decades, various novel alloying strategies have been developed to overcome the limitations of traditional single‐principle alloy design concepts, leading to the emergence of a new class of materials known as high‐entropy alloys (HEAs).^[^ ^1^, ^2^, ^3^, ^4^, ^5^, ^6^, ^7^, ^8^, ^9^, ^10^, ^11^, ^12^, ^13^, ^14^, ^15^ ^]^ In HEAs, various alloy components do not occupy absolute dominant positions, and the overall alloy performance is determined by the synergistic interplay between the characteristic properties of the constituent components.^[^ ^2^, ^3^ ^]^ Expansion of the compositional space in alloy design has led to the discovery of materials with unprecedented and compelling properties.^[^ ^1^, ^4^ ^]^ For example, a CrMnFeCoNi single‐phase face‐centered cubic (FCC) solid‐solution HEA was reported to exhibit exceptional damage tolerance, ultrahigh tensile strength, and remarkable fracture toughness.^[^ ^5^ ^]^ In addition, the incorporation of highly plastic intermetallic nanoparticles into a multicomponent FeCoNiAlTi alloy has enabled the development of super‐HEAs with high strength and plasticity.^[^ ^12^ ^]^ Although numerous research avenues are opening up this dynamic field, efforts have largely focused on enhancing the mechanical properties and exploring novel physical mechanisms. In contrast, few studies have focused on functional properties such as magnetic behavior.^[^ ^16^, ^17^, ^18^, ^19^, ^20^ ^]^

Recently, uniformly dispersed paramagnetically ordered nanoparticles were constructed in a multicomponent FeCoNiAlTa matrix, which hindered dislocation movement and increased the overall material strength and ductility. Notably, the small size of these nanoparticles, which is smaller than the domain‐wall width, minimizes the domain‐wall pinning effect, achieving excellent mechanical properties and soft magnetic properties in the bulk alloys.^[^ ^16^, ^17^, ^19^ ^]^ Although existing ferromagnetic HEAs exhibit excellent toughness and plasticity characteristics along with reasonable soft magnetic properties, their relatively high coercivities prevent them from competing with traditional soft magnetic materials. Meanwhile, amorphous alloys (AMAs) have emerged as promising alternatives owing to their superior soft magnetic properties, including their low coercivities (*H*_c_), high saturation magnetic flux densities (*B*_s_), high permeabilities (*µ_e_*), and minimal saturation magnetostriction,^[^ ^21^, ^22^, ^23^, ^24^, ^25^, ^26^, ^27^, ^28^ ^]^ which render them more advantageous than traditional Si steel sheets and crystalline magnetic materials. Notably, the rapid development of next‐generation electronic information technologies has intensified the demand for soft magnetic ribbons capable of maintaining long‐term stable operations in high‐load scenarios. Unfortunately, neither disordered AMAs nor ordered HEAs satisfy the dual requirements of excellent soft magnetic properties and mechanical properties. This pressing need underscores the urgency to design and develop new soft magnetic materials that can integrate these characteristics.

Thus, in the present study, a novel transitional structure that synergistically combines the advantages of HEAs and AMAs was designed. It is expected that the HEAs will contribute to high configuration entropies and the ability to construct micro‐nano crystal structures that facilitate plastic deformation, whereas AMAs should provide disordered structures and exhibit low magnetic anisotropy. Initially, Si and B were doped into the FeCoNiAlTa HEA matrix to form a fully amorphous precursor. Subsequently, an order‐modulation strategy was employed to construct the transitional structure via a controlled and short annealing process near the crystallization temperature (*T_x_*). This process yielded Fe_26_Co_25_Ni_25_Al_3_Ta_1_Si_2_B_18_ amorphous‐nanocrystalline transition state (ANTS) ribbons, which exhibited remarkable toughness and were capable of bending 180° at any angle without fracture. The Fe_26_Co_25_Ni_25_Al_3_Ta_1_Si_2_B_18_ ANTS ribbons exhibited outstanding soft magnetic properties, including an ultra‐low *H*_c_ of 0.3 A m^−1^, a high *µ_e_* of up to 13 200 at 1 kHz, and a *B*_s_ of up to 1 T. These properties make the ANTS ribbons highly suitable for applications in electronic power‐functional devices operating under complex conditions. This study also aimed to establish a foundational framework for the future development of high‐performance soft magnetic HEAs and offer new insights into the design of advanced materials that simultaneously achieve mechanical robustness and excellent magnetic properties.

## 2 Results and Discussion

### 2.1 Component Design Concept

**Figure 1:**
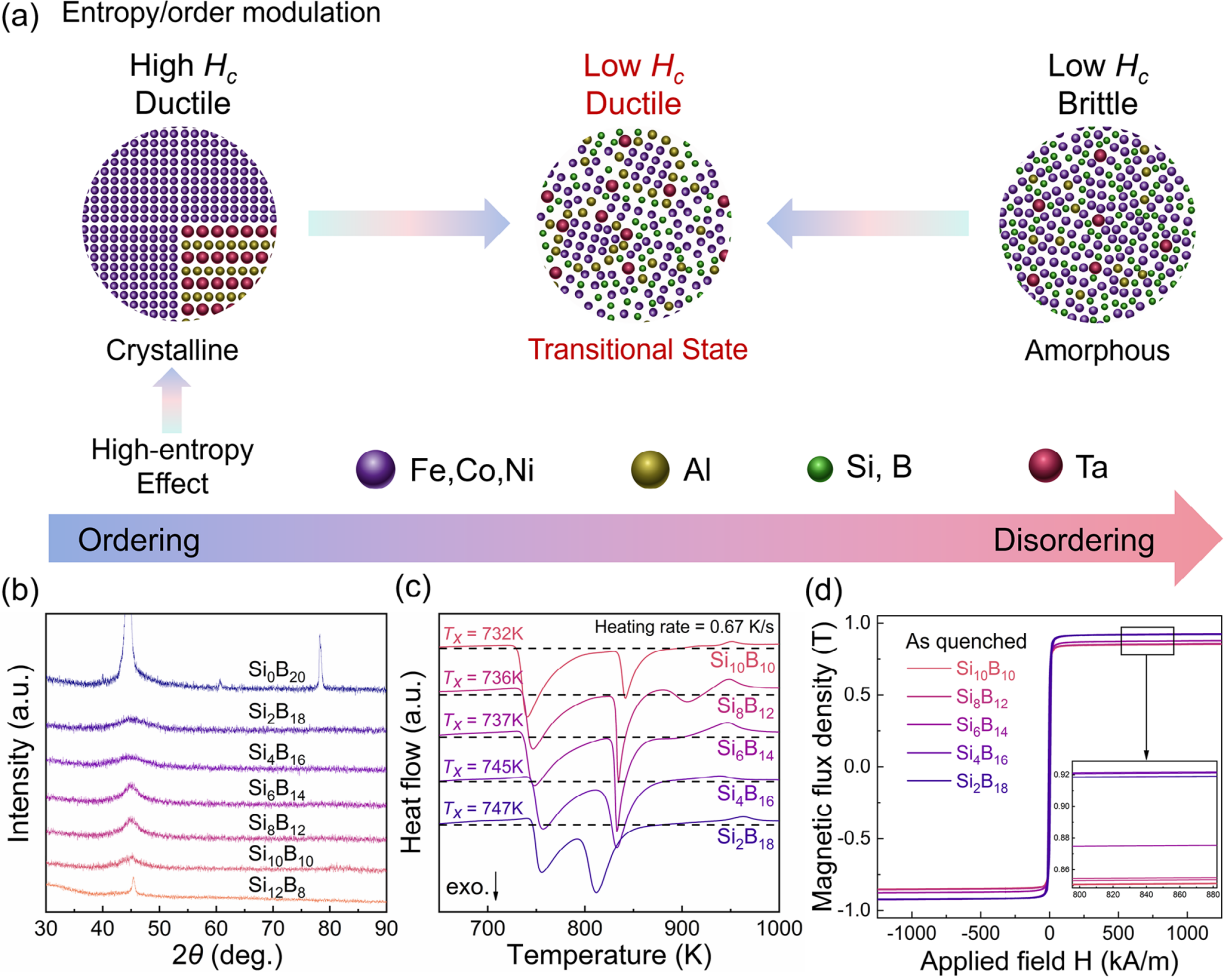
a) Schematic of the component structure design strategy. b) XRD patterns for the various prepared specimens. c) DSC curves for the various prepared HEAAs. d) Room‐temperature hysteresis loops recorded for the various prepared HEAAs.

The component design strategies shown in **Figure** **1**a are adopted according to the definitions of HEAs with equal or nearly equal atomic percentages. In order to overcome the bottleneck of insufficient mechanical, especially the ability of plastic deformation, nearly equal atomic ratios of Fe, Co, and Ni were used in the alloy matrix. It has been widely demonstrated that an appropriate amount of Al atoms can alter the electron density and local valence bonds of materials, promoting the formation of partial body‐centered cubic (BCC) structures and improving their ability to resist magnetic degradation.^[^ ^29^, ^30^, ^31^, ^32^, ^33^ ^]^ Consequently, mechanical robustness and soft magnetic properties can be balanced by adding Al. Additionally, Ta atoms were introduced to inhibit diffusion and realize the controllable precipitation of fine nanocrystals. According to the three principles proposed by Inoue for the formation of AMAs,^[^ ^34^ ^]^ metal‐like elements, such as Si and B, can be added to improve the glass‐forming ability (GFA) of a material. However, the addition of excess B leads to the precipitation of FeB during the nanocrystallization process, which degrades the soft magnetic properties. Thus, detailed experiments were performed to control the Si and B contents and enhance both the GFA and soft magnetic properties of the resulting alloy. Moreover, considering that the vacancies in the three‐dimensional (3D) atomic orbitals of ferromagnetic atoms contribute to the amplitude of the local magnetic moment and are filled by the outer electrons of the metalloid‐like elements in ferromagnetic alloys,^[^ ^21^, ^35^ ^]^ the amplitude of the local magnetic moment can be maximized by introducing an appropriate ratio of Si and B.

The alloys prepared in this study followed the general formula Fe_26_Co_25_Ni_25_Al_3_Ta_1_Si_20−x_B_x_ (x = 0, 2, 4, 6, 8, 10, 12, 14, 16, 18, and 20 at.%). The X‐ray diffraction (XRD) patterns of the free sides of the spun alloy ribbons are shown in Figure 1b, where broad diffraction peaks are observed for x = 10, 12, 14, 16, and 18 at.%, indicating that these alloy ribbons are completely amorphous. In contrast, for x = 8 and 20 at.%, sharp peaks appeared, corresponding to a crystalline phase, and at x = 0, 2, 4, and 6 at.%, the ribbons were severely crystallized in the as‐quenched (AQ) state, resulting in hardness and brittleness. With an increasing B content, the diffraction angle of the amorphous halo peak increased, indicating that the distance between the nearest‐neighbor atoms decreased. This suggested the formation of an amorphous phase with denser atomic packing, which also gradually increased under these preparation conditions. Figure 1c shows the differential scanning calorimetry (DSC) trajectories of the amorphous ribbons, where two to three stages of crystallization can be observed. At higher B contents, the initial exothermic temperature increased, leading to a gradual increase in *T_x1_* and a decrease in *T_x2_*. The observed increase in *T_x1_* indicates that the addition of B enhances the thermal stability of the amorphous ribbon, whereas the decrease in *T_x2_* may be due to the increased precipitation tendency caused by the change in alloy composition of the remaining amorphous phase after the initial precipitation.^[^ ^36^, ^37^, ^38^ ^]^ Figure 1d shows the room‐temperature hysteresis loops of the five amorphous ribbons, all of which exhibit typical soft hysteresis characteristics. With an increase in the B content, the value of *B*_s_ increased gradually, reaching the highest value of 0.92 T for the Fe_26_Co_25_Ni_25_Al_3_Ta_1_Si_2_B_18_ alloy. In this alloy system, the substitution of B for Si reduces the average atomic radius of the amorphous alloy, thereby enhancing the ferromagnetic exchange coupling and consequently increasing the saturation magnetic flux density. However, excessive B replacement compromises GFA. When the B content reaches 20 at.%, the Fe_26_Co_25_Ni_25_Al_3_Ta_1_B_20_ alloy cannot form a fully amorphous state, indicating that B substitution optimizes *B*_s_ only up to this limit. This ribbon composition was selected for use in subsequent experiments because of its superior thermal stability and higher *B*_s_ value compared with the other compositions. Moreover, this composition is critical for the transition from high‐entropy amorphous alloys (HEAAs) to HEAs, theoretically facilitating the development of a transition‐state structure to obtain excellent and comprehensive soft magnetic properties.

### 2.2 Structural Design Strategy

**Figure 2:**
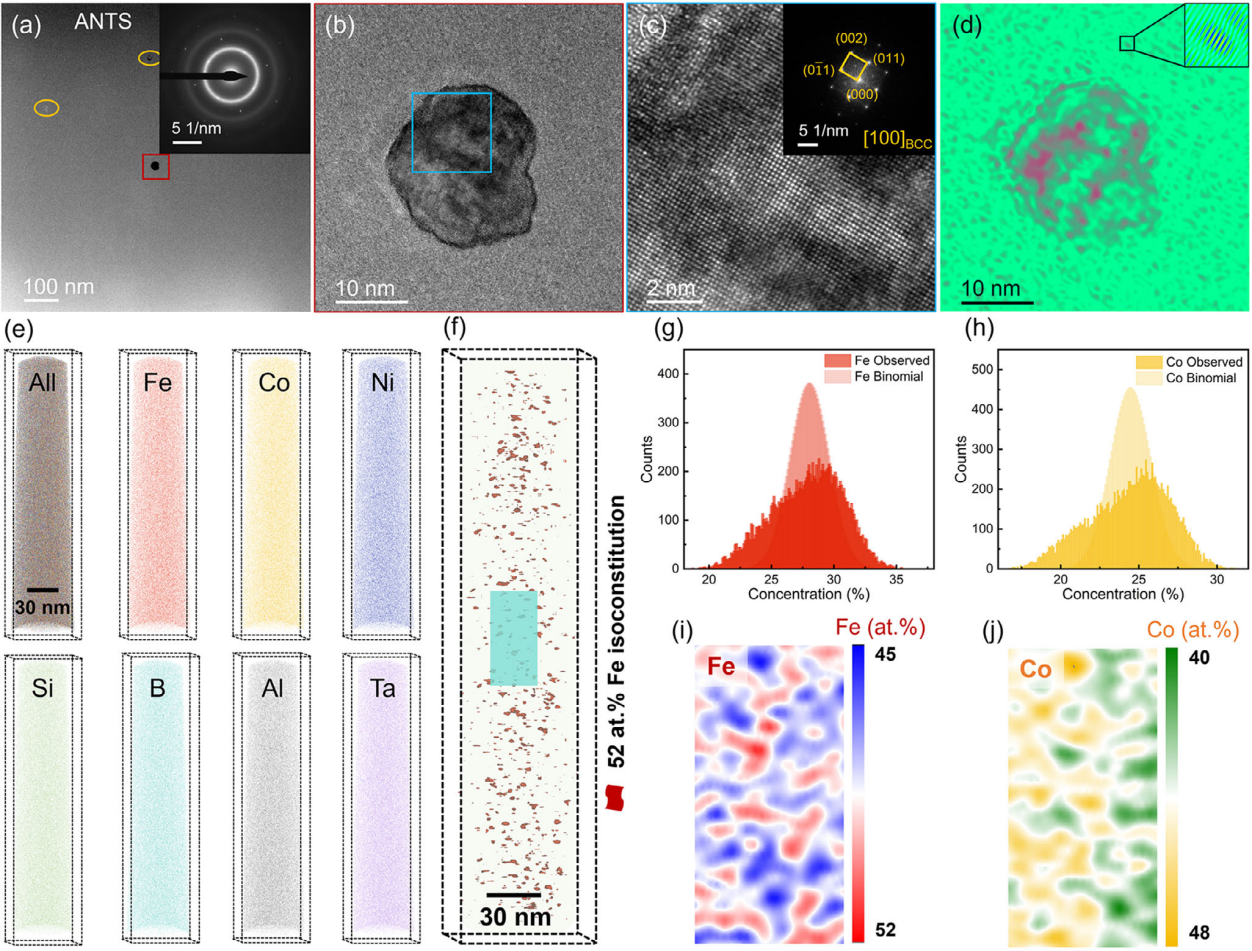
Microscopic structures in the ANTS states: a) TEM image of the annealed sample after 2 min of heat treatment at 743 K, showing the microstructures of the nanocrystalline grains sparsely distributed within the amorphous matrix. The inset shows the corresponding SAED pattern. b) Enlarged image of the area indicated by the red square in panel a). c) Enlarged image of the area indicated by the blue square in panel b). The inset shows the corresponding FFT pattern. d) IFFT pattern in panel b). e) Atomic probe analysis showing the tomography of the ANTS ribbon. f) A surface bearing 52 at.% Fe is used to highlight the outline of the crystal‐like orders. g) Binomial distribution of the experimental data in comparison with the theoretical binomial random distribution of Fe. h) Binomial distribution of the experimental data in comparison with the theoretical binomial random distribution of Co. i) 2D projection of the Fe concentration slice acquired from panel f). j) 2D projection of the Co concentration slice acquired from panel f).

In this study, an order‐modulation design strategy was employed to build the transition structure.^[^ ^26^, ^39^, ^40^, ^41^, ^42^ ^]^ This structure falls between an amorphous state and an ordered crystal state, and it possesses a specific order that is not based on nanocrystals. The Fe_26_Co_25_Ni_25_Al_3_Ta_1_Si_2_B_18_ amorphous ribbons were annealed at various temperatures and for various durations, and their microstructures were examined using high‐resolution transmission electron microscopy (HRTEM) and selected‐area electron diffraction (SAED). The HRTEM images presented in Figures S2a,b (Supporting Information) show that crystal precipitation did not occur. In addition, the SAED image shows only a diffuse halo, and the atomic distribution in the high‐magnification bright‐field image is represented by both short‐ and long‐range disorder. This result indicates that both the AQ and relaxed‐state (RS) ribbons maintained an amorphous structure. As shown in **Figure** **2**a, small quantities of BCC nanocrystals precipitated (Figures 2c), which differs from the precipitation of fine uniform nanocrystals with a volume percentage of >70% under the traditional Fe‐based amorphous ribbon annealing treatment.^[^ ^26^, ^43^, ^44^, ^45^ ^]^ This is expected to affect the strength and toughness, while resulting in extremely low *H*_c_ values. Moreover, from the inverse fast Fourier transform (IFFT) of the HRTEM image (Figure 2b,d), numerous crystal‐like orders (<2 nm) were uniformly distributed in the amorphous matrix around the nanocrystals, which were assigned to ANTS. Furthermore, elemental segregation during the nucleation process was evidenced by 3D atom probe tomography (3D‐APT). All the elements were detected in ANTS, as shown in Figure 2e. Additionally, Figure 2f shows the 3D atomic distribution with a 52% Fe iso‐constitution surface of Fe, in which numerous Fe‐rich nanograins (<2 nm) can be clearly observed, consistent with the cluster size determined via TEM. The frequency‐distribution analysis confirmed the compositional segregation of Fe and Co (Figure 2g,h, respectively), while the 2D contour plots in Figure 2i,j confirm the heterogeneous distribution of these elements. These results indicate Fe and Co enrichment in the ANTS. Furthermore, the ribbons shown in Figure S2c (Supporting Information) possess numerous irregularly shaped nanocrystals evenly distributed within the amorphous matrix, reaching a volume fraction close to that of a typical Fe‐based nanocrystalline alloy obtained via heat treatment of amorphous ribbons. This corresponds to the amorphous‐nanocrystalline state (ANS), and it can be seen from Figure S2e,f (Supporting Information) that the precipitated nanocrystalline phase also corresponds to a BCC structure.

### 2.3 Magnetic Properties

**Figure 3:**
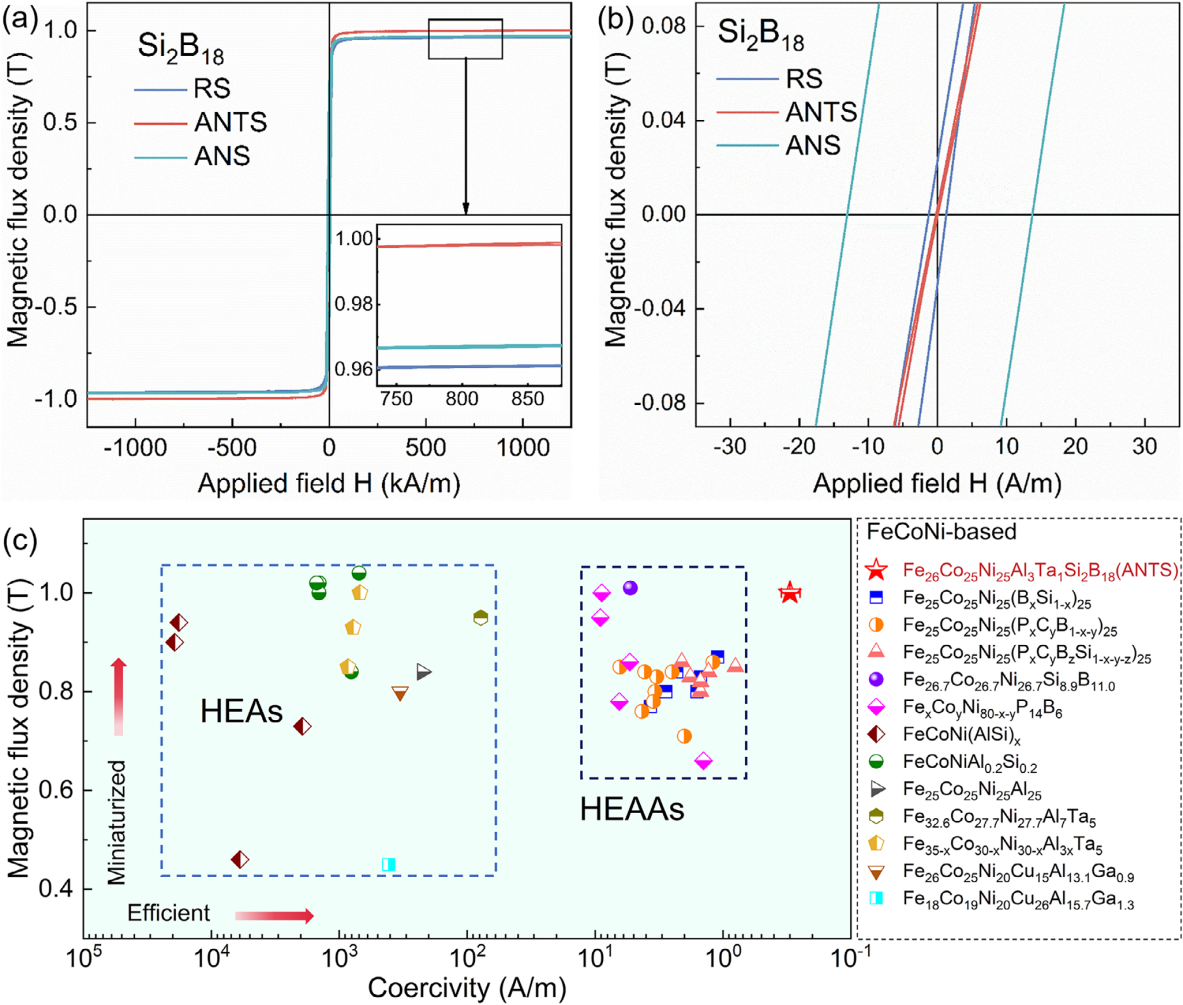
Soft‐magnetic properties of the various states and materials: a) Hysteresis loops in the different states, b) *B–H* loop amplification, c) *B*_s_ and *H*_c_ values of the FeCoNi‐based HEAs, previous HEAAs, and new HEAAs.^[^ ^29^, ^46^, ^47^, ^48^, ^49^, ^50^, ^51^, ^52^, ^53^ ^]^

The effects of the RS, ANTS, and ANS systems on the soft magnetic properties of the Fe_26_Co_25_Ni_25_Al_3_Ta_1_Si_2_B_18_ amorphous ribbons are shown in **Figure** **3**. Figure 3a,b presents the corresponding *H*_c_ and *B*_s_ values, respectively, of the different states. For the ANTS system, the highest *B*_s_ value of 1 T was achieved, along with the lowest *H*_c_ value of 0.3 A m^−1^, representing a breakthrough in the trade‐off between *B*_s_ and *H*_c_. This result also indicates the improvement of the saturation magnetization intensity of the ANTS sample after annealing is mainly due to the precipitation of numerous Fe‐rich nanograins through the construction of an ANTS, significantly improving the soft magnetic properties of the AMA. The decrease in *H*_c_ was attributed to the reduction in the magnetoelastic anisotropy caused by the release of residual internal stress during the casting process, along with the weakening of the magnetic‐domain pinning effect. When the annealing temperature was higher than *T_x1_* and the amorphous ribbon precipitated to generate uniform nanocrystals, *H*_c_ rapidly decreased to more than tens of A/m, reaching a value similar to that of traditional Fe‐based nanocrystals. Notably, the requirements for nanocrystallization are extremely strict, and the time interval from nucleation to size degradation is only tens of seconds. The *B*_s_ and *H*_c_ values of the previously reported FeCoNi‐based HEAs and HEAAs, along with those of the current ANTS, are presented in Figure 3c. The systems prepared in this study exhibited higher *B*_s_ values and lower *H*_c_ values than those reported in the literature. Furthermore, the ANTS ribbon exhibits an extraordinarily high resistivity, reaching 198 µΩ·cm, which significantly exceeds those of soft magnetic materials reported in previous studies, as shown in Figure S5 and Table S1 (Supporting Information). Therefore, the ANTS ribbon is anticipated to exhibit a significant advantage with regard to eddy current loss. Moreover, Figure S6 (Supporting Information) shows the variation curves of *µ_e_* for the amorphous ribbons in the different states, where it is apparent that although the RS can effectively increase *µ_e_*, the ANTS leads to a more significant increase, reaching 13 200 at 1 kHz. This maximum *µ_e_* can be attributed to the fact that a higher annealing temperature is conducive to the release of internal stress, a reduction of the pinning effect that affects the movement of magnetic domains, and the formation of sparse and fine nanocrystals surrounded by abundant crystal‐like orders. Ultimately, these factors inhibit magneto‐crystalline anisotropy and reduce saturation magnetostriction.

### 2.4 Mechanical Properties

**Figure 4:**
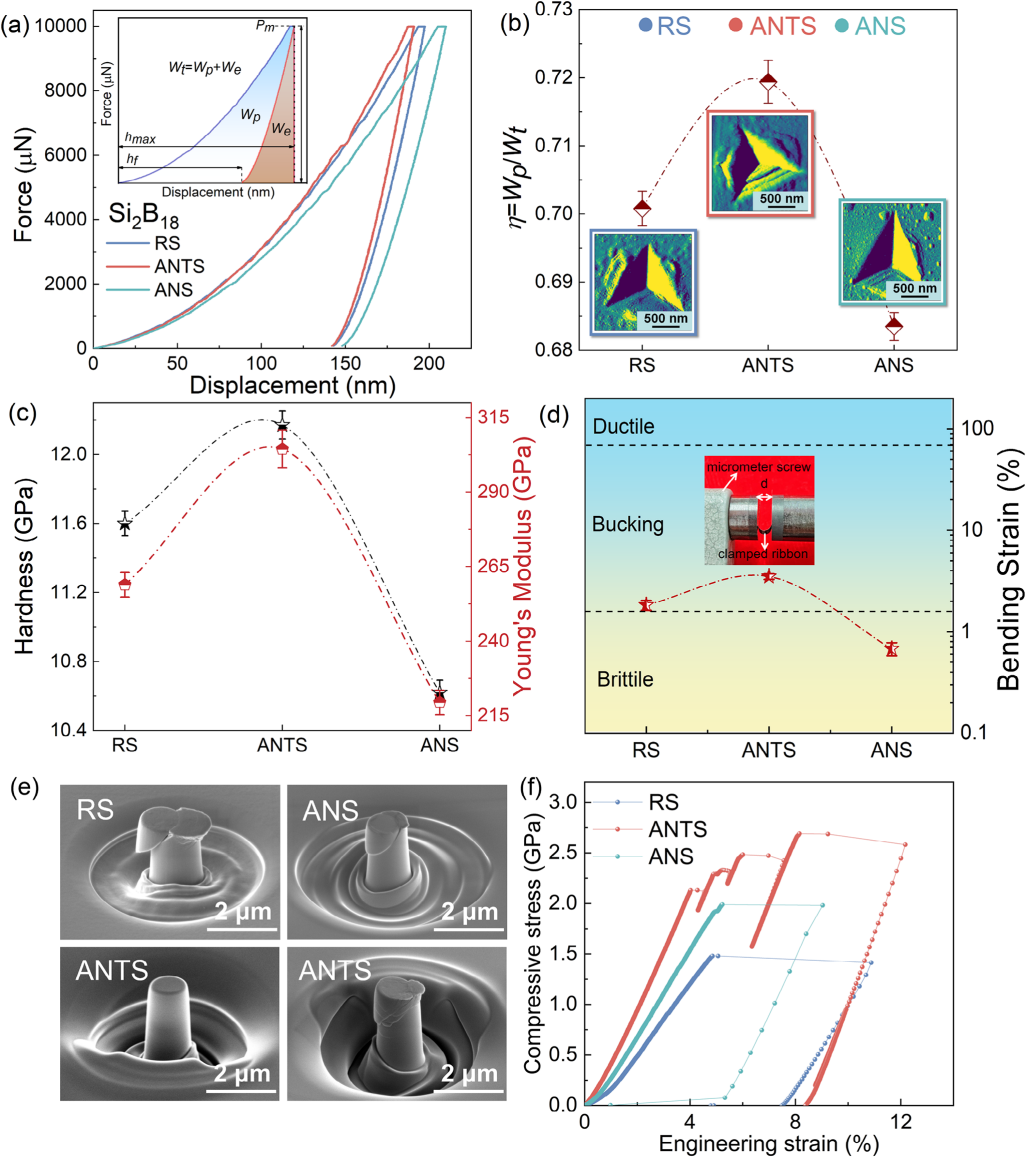
Mechanical properties of the various states: a) nanoindentation curves; b) retrospective plot of the indentation amplitude as observed by plastic work and AFM; c) hardness values and Young's moduli; d) bending strains and specific failure modes; e) morphology images after compression of ANTS, RS, and ANS ribbons; f) engineering stress‐strain curves recorded for the pillars with a diameter of 1 µm and height of 2 µm.

In addition to their soft magnetic properties, the mechanical properties of the prepared alloys were evaluated. **Figure** **4**a shows the load‐displacement curves recorded for the Fe_26_Co_25_Ni_25_Al_3_Ta_1_Si_2_B_18_ amorphous ribbons at a maximum load of 10 000 µN. These curves demonstrate the occurrence of plastic deformation in the material after indentation, as the curves of the different states did not return to their origins after the removal of the load. Additionally, upon the initial application of a load, the material underwent elastic deformation, and with an increase in the load, plastic deformation gradually occurred. The unloading curves indicate the recovery of the elastic deformation, wherein the maximum indenter penetration depth (*h_max_*) is generated at the maximum applied load, and the final depth (*h_f_*) is the depth of the residual indentation when the indenter is removed (inset, Figure 4a).^[^ ^6^, ^54^, ^55^ ^]^ To quantify the microscopic elastoplastic behavior of the samples, the approach proposed in^[^ ^56^ ^]^ was employed, wherein the areas under the indentation loading and unloading curves were measured. Considering that *W_t_* is the total work of the indentation (area under the loading curve), *W_e_* is the work recovered during unloading (area under the unloading curve), *W_p_* is the plastic work of the indentation, and *W_p_*/*W_t_* is the indentation toughness, the results shown in Figure 4b indicate significantly higher values for the ANTS than for the other states.

Atomic force microscopy (AFM) was employed to visually observe the surface topography of the samples after indentation in different states (Figure 4b). For ANTS, a series of locally circular shear bands were observed in the accumulation zone around the indentation, wherein a larger number of shear bands were observed than in the other states. This is consistent with the above results, indicating that the ANTS ribbons exhibit excellent microscopic toughness characteristics in this ideal transition state. Furthermore, Figure 4d illustrates the macroscopic bending behavior of the ribbon, where the sample was sandwiched between two plate surfaces and the spiral micrometer was gradually adjusted to a shorter distance, increasing the bending strain of the ribbon. Three ribbon response modes were considered: toughness, buckling, and brittle fracture. Brittleness was defined the ribbon breaking completely during the flexure test. In contrast, the buckling ribbon is not completely broken but is unable to withstand further plastic deformation, while the ductile ribbon exhibits no fracture or buckling behavior and remains intact.^[^ ^57^, ^58^, ^59^, ^60^ ^]^ The bending strain at break or buckling was calculated using the following formula (1)^[^ ^60^ ^]^

(1) $$\varepsilon_{f/b}=\frac{t}{d-t}$$

where *t* represents the thickness of the ribbon, and *d* represents the distance between the two surfaces. When *d* = 2*t*, the ribbon bends 180°. As shown in Figure 4d, the bending‐strain capacity of the ANTS was significantly higher than that of the RS and ANS systems, demonstrating a good plastic deformation ability, which differs from the hard and brittle characteristics of traditional Fe‐based amorphous ribbons after high‐temperature annealing. Figure 4c shows the hardness (*H*) and Young's modulus (*E*) of the amorphous ribbon as functions of the annealing state. *H* and *E* were calculated using the conventional depth‐sensing indentation method described by Oliver and Pharr,^[^ ^54^ ^]^ according to Equations (2) and (3):

(2) $$H=\frac{P_{m}}{A(h_{c})}$$

(3) $$E=\frac{\sqrt{\pi }}{2\beta }\frac{S}{\sqrt{A(h_{c})}}$$

where *P_m_* represents the maximum load, *A*(*h_c_*) represents the contact area of the Berkovich indenter, *β* is a constant related to the geometry of the indenter, and *S* represents the contact stiffness determined by the initial unloading slope, which determines the contact stiffness of the Berkovich indenter as 1.034. In general, *E* represents the intrinsic property of a material that depends on the strength of the bonds between the constituent atoms. In contrast to crystalline alloys, AMAs are considered to exist in a non‐equilibrium state with a disordered structure. The strength of the bonds between the constituent atoms varies with the degree of order in the microstructure, which is not constant.^[^ ^6^ ^]^ The annealing process changed the intrinsic structure of the amorphous ribbon, giving mean values of 11.6, 12.17, and 10.62 GPa for *H* and mean values of 258.9, 304.4, and 219.4 GPa, respectively, for *E*, as shown in Figure 4c. Both the *H* and *E* values of ANTS were higher than those of the other states, indicating that the material had increased resistance to deformation. In addition, owing to the obvious high‐entropy effect, the *H* and *E* values of the ANTS ribbons were higher than those of the traditional Fe‐ and Co‐based amorphous ribbons. This can also be attributed to the special slow diffusion effect observed in materials with high mixed entropies, which hinders atomic movement during plastic deformation and significantly reduces the Gibbs free energy of the system, resulting in stronger cohesion or atomic bonding.^[^ ^61^, ^62^ ^]^ Microcompression tests were performed on micropillars fabricated from the ribbons. Figure 4e shows the morphology images recorded after pillar compression. The RS and ANS pillars exhibited features corresponding to brittle fracture and slippage, whereas the ANTS pillars demonstrated multiple shear bands. Figure 4f presents the compressive engineering stress‐strain curves of pillars with a diameter of 1 µm and height of 2 µm for the different states. The RS and ANS ribbons exhibited yield strengths of 1.5 and 2.0 GPa, respectively, without plasticity, indicating their intrinsic brittleness.^[^ ^63^, ^64^, ^65^, ^66^ ^]^ In contrast, the ANTS ribbon demonstrated a yield strength of 2.1 GPa with multiple enhancements, reversing the phenomenon observed in amorphous and nanocrystalline alloys, wherein the breakage of only one main shear band occurs.

### 2.5 Mechanisms of Magnetization

Although HEAs demonstrate good macroscopic tensile toughness, their high values of *H*_c_ lead to increased hysteresis loss and energy consumption, significantly limiting their application as soft magnetic materials. Traditional AMAs can effectively reduce *H*_c_, but the embrittlement characteristics caused by high‐temperature annealing render their mechanical load capacities insufficient for use in electronic devices. However, the Fe_26_Co_25_Ni_25_Al_3_Ta_1_Si_2_B_18_ alloy developed in this study not only demonstrated good toughness exceeding that of traditional AMA ribbons but also exhibited an ultra‐low *H*_c_ of 0.3 A m^−1^. Notably, this value is lower than those of all previously reported HEAs. In particular, the ANTS of Fe_26_Co_25_Ni_25_Al_3_Ta_1_Si_2_B_18_ achieved a good combination of high toughness and ultra‐low *H*_c_, as shown in Figure S9 (Supporting Information). This structure differs from that of a typical Finemet amorphous‐nanocrystalline alloy, wherein the addition of Cu promotes nucleation and the inclusion of Nb inhibits nanocrystalline growth.^[^ ^23^, ^26^ ^]^ In contrast, the addition of small amounts of Al and Ta to replace some ferromagnetic elements in the current system produces an ideal transition structure between amorphous and traditional nanocrystals. Moreover, controlling the Si and B content maximizes the amplitude of the local magnetic moment and overcomes the mutually exclusive relationship between the mechanical and magnetic properties.^[^ ^21^ ^]^

**Figure 5:**
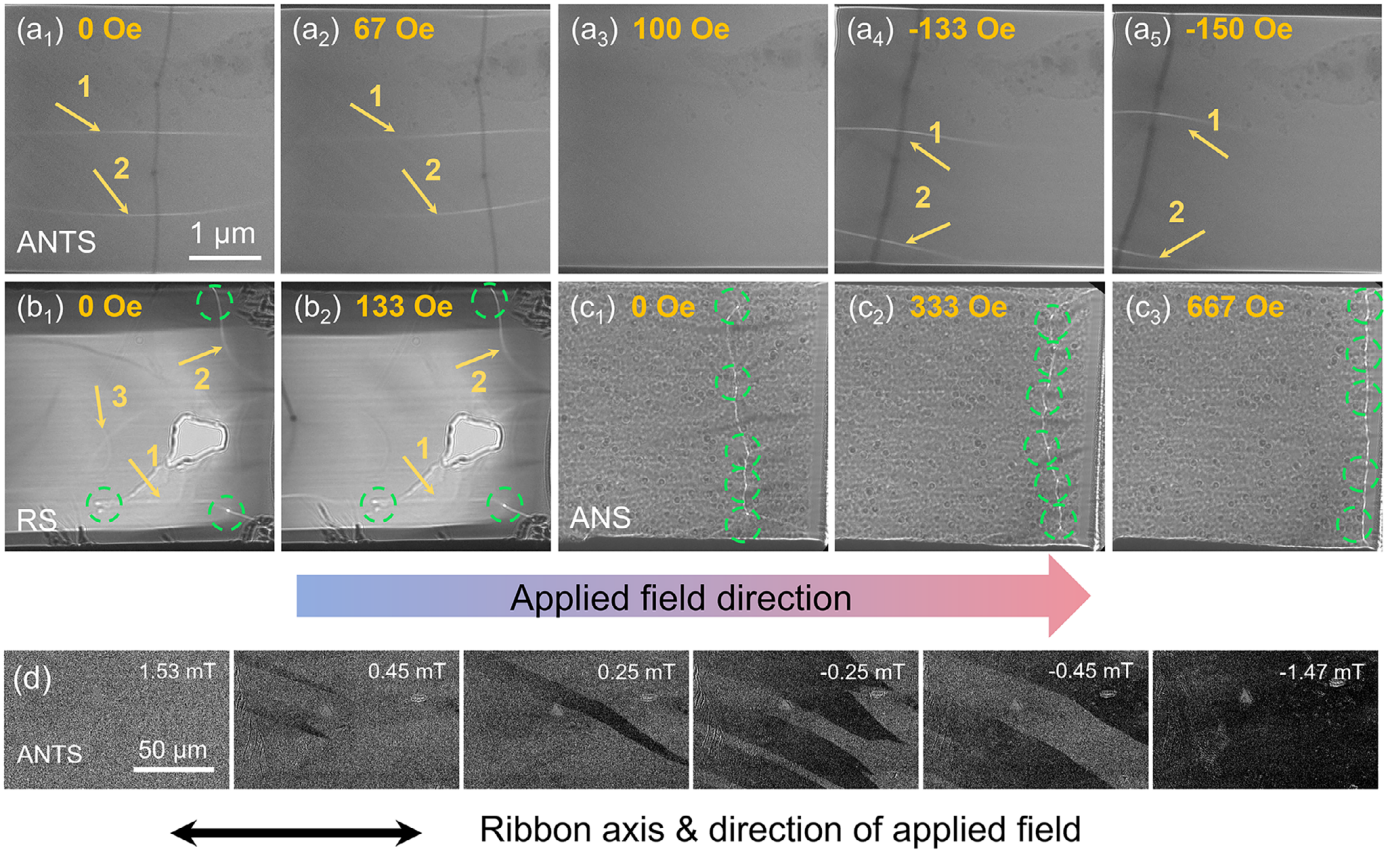
a) LTEM images recorded for the ANTS. b) LTEM images recorded for the RS. c) LTEM images recorded for the ANS. d) Magneto‐optical Kerr subset images showing the evolution of the magnetic‐domain structure for the ANTS. The yellow arrows labeled 1, 2, and 3 in panels a) and b) represent the magnetic‐domain walls, while the green circles in panels b) and c) represent the pinning points.

To explore the origin of this excellent soft magnetic performance, the magnetic‐domain structures were examined using Lorentz TEM (LTEM), as shown in **Figure** **5**a,c. The pinning effect of the ANTS ribbons was not significant. As the magnetic field increased, domain walls 1 and 2 moved smoothly, and they disappeared when the magnetic field reached 100 Oe (Figure 5a_3_). Subsequently, when the direction of the magnetic field is reversed, the domain walls move smoothly in the opposite direction, as shown in Figure 5a_4_,a_5_. For the RS ribbon (Figure 5b), as the magnetic field increased from 0 to 133 Oe, domain wall 3 disappeared, whereas domain walls 1 and 2 remained pinned at their initial positions. However, a larger number of pinning points were observed in the ANS ribbon, where the domain walls were strongly pinned. When the magnetic field was increased to 667 Oe, the domain walls were only weakly triggered. Subsequently, magneto‐optical Kerr microscopy was employed to analyze the magnetic‐domain structures of the ribbons and to observe the magnetic‐domain movement in the samples in different states. Figure 5d and Figure S8 (Supporting Information) show Kerr subset diagrams representing the evolution of the domain structure. Initially, the Kerr image of the ribbon was magnetized to saturation along the positive direction. As the strength of the counter magnetic field increased, the domain wall emerged from the middle and gradually extended along the entire width. Subsequently, the domain wall moved approximately perpendicular to the ribbon and gradually disappeared. The change in the magnetic field required for the entire process was calculated from the appearance of the domain wall to its disappearance. As shown in Figure 5d, the minimum magnetic‐field spacing of the ANTS system was 3 mT, indicating the smallest level of magnetic‐domain motion resistance among the various states. Owing to the stress concentration caused by the residual internal stress, the movement of the magnetic domain was more challenging during the magnetization process, and the magnetic domain of the AQ ribbon was irregular, uneven, and mixed with typical labyrinthine stress fields, as shown in Figure S8a (Supporting Information). In contrast, it can be seen from Figure S8b (Supporting Information) that the prolonged annealing process promoted the release of residual stress. In this case, the orientation of the static domains was highly consistent, and the domain width was more uniform, resulting in a decrease in the magneto‐elastic anisotropy, which is conducive to the inversion of the magnetic domains. As shown in Figure 5d, the ANTS system not only eliminated the residual stress in the ribbon which reduced the magneto‐elastic anisotropy, but also constructed a sparse and fine amorphous‐nanocrystalline structure, which make the ribbon have lower magneto‐crystalline anisotropy. This increased the local atomic mean magnetic moment and generated a wider and smoother regular‐fringe magnetic domain. However, observing the motion of the magnetic domain (Figure S8c, Supporting Information) revealed that the domain was pinned at the grain, which was mainly due to high‐temperature annealing, leading to an excess of the ideal amorphous transition state, subsequent rapid nucleation, and lattice defect formation. Consequently, the interaction with the magnetic‐domain wall and the loss of domain‐wall motion led to the rapid deterioration observed for *H*_c_.

## 3 Conclusion

In this study, the design concepts of HEAs and AMAs were combined to prepare Fe_26_Co_25_Ni_25_Al_3_Ta_1_Si_2_B_18_ soft magnetic ANTS ribbons. This was achieved by incorporating Si and B into the FeCoNiAlTa matrix, which significantly enhanced the soft magnetic properties of the material. Using an order‐modulation strategy, the precipitation of fine nanocrystals was induced. The sparse nanocrystals were surrounded by numerous crystal‐like orders within an amorphous matrix under controlled high‐temperature annealing conditions. This process resulted in the formation of an ideal ANTS. The resulting material exhibited exceptional toughness and ultra‐low *H*_c_, achieving an effective combination of excellent soft magnetic properties and outstanding mechanical performance. This balance was mainly attributed to the rapid release of stress in the ribbon over a short annealing period as well as the precipitation of a small volume fraction of nanocrystalline phases. These characteristics collectively contributed to significant reductions in both the magneto‐crystalline anisotropy and magneto‐elastic anisotropy. Additionally, the local atomic magnetic moment was enhanced, whereas the domain‐wall pinning effect was minimized. This study provides a novel approach for the development of high‐performance soft magnetic HEAs for demanding applications.

## 4 Experimental Section

### Material Preparation

Alloy ingots with a nominal composition of Fe_26_Co_25_Ni_25_Al_3_Ta_1_Si_20−x_B_x_ (x = 0, 2, 4, 6, 8, 10, 12, 14, 16, 18, and 20 at.%) were prepared via arc‐melting of the constituent elements (>99.99 wt.% purity) in an Ar atmosphere. Each ingot was flipped and remelted at least five times to achieve chemical homogeneity. Subsequently, ribbons were prepared from the alloy ingots using the single‐roller melt‐spinning technique. The precursor amorphous ribbon with a thickness of 23 µm and a width of ≈1 mm was prepared in a 1 × 10^−3^ Pa high‐vacuum argon atmosphere with a Cu wheel linear velocity of 40 m s^−1^. The size of the quartz tube nozzle used in the single‐roller melt‐spinning process was ≈1 mm, the distance between the nozzle and the Cu roller was ≈0.4 mm, and the injection pressure was 0.02 MPa. Archimedes’ method was used to measure the density of each alloy ingot, and a minimum of five tests were performed for each batch. The density fluctuated between 7.79 and 7.86 g cm^−3^. Considering the measurement error, the minimum density value was selected to calculate the *B*_s_ value. The ribbon annealing process was completed using a self‐developed magnetic field compound heat treatment system (NMS‐CCRCL‐II), which used resistance wires to preheat the furnace chamber to the target temperature. After the temperature was stabilized, the samples stored in vacuum (<1 × 10^−3 ^Pa) were placed in the specified position of the furnace chamber and removed when the desired annealing time was reached. (“ANTS” represents the sample annealed at 743 K for 2 min, “ANS” represents the sample annealed at 743 K for 4 min, and “RS” represents the sample annealed at 673 K for 60 min)

### Microstructure and Thermodynamic Testing

The phase structures of the ribbon samples were analyzed using XRD (Bruker D8 Advance) with Cu Kα irradiation. The *T_x_* values of the samples were determined using DSC (Netzsch 404 F3) at a constant heating rate of 0.67 K s^−1^. The ribbons were thinned using an ion‐thinning apparatus (Gatan 695), and the nanostructures of the samples were characterized using HRTEM (FEI Talos F200). In situ dynamic observations of the magnetic viscosity were performed using a Lorentz transmission electron microscope (FEI Talos F200) and a horizontal magnetic in situ sample holder prepared in‐house. The nanoscale elemental distribution was investigated using 3D‐APT with a local electrode atom probe (Cameca Instruments LEAP 4000X HR). The needle‐shaped specimens required for APT were fabricated using a focused ion beam (FIB)‐based lift‐out approach and annular milling (FIB‐SEM, FEI Scios).

### Evaluation of Magnetic Properties

The *H*_c_ value of each ribbon was measured using an alternating current *B‐H* analyzer (IWATSU, SY‐8218), while the *M_s_* and *T_c_* values were determined using a magnetic measurement system (MPMS, Quantum Design), and *µ_e_* was obtained using an impedance analyzer (Agilent 4294A). The magnetic domains of the ribbons were observed and analyzed using a Magneto‐optical Kerr microscope (EM Kerr).

### Evaluation of Mechanical Properties

The hardness and elastic moduli of the ribbon samples were analyzed using a nanoindentation device (Bruker TI980), and the ribbon toughness characteristics in different states were analyzed using the obtained nanoindentation curve. The indentation height distribution was observed using a high‐resolution atomic force microscope (Cipher S AFM Microscope, Oxford Instruments, Asylum Research). Ribbon micropillars were fabricated using an FIB‐based approach (Helios 5UX). The aspect ratio (height/diameter) of each micropillar was set to 2, and the diameter was 1 µm. Microcompression tests were conducted at room temperature in the displacement‐control mode and at a strain rate of 5×10^−3^ s^−1^, using the low‐load mode of the nanoindenter and a flat punch diamond tip (Bruker TI980). Each test was conducted using three pillars.

### Statistical Analysis

Quantification was described in the method details and figure legends. Statistical analysis was completed using Origin 2021 and statistical significance is exhibited in the relevant figures and figure legends. The number of samples was shown and described in the figure legends. Error bars represent the standard error of the standard deviation as indicated in the figure legend, and the statistical tests were defined in the figure legends. Experimentalists remained blinded to experimental conditions whenever possible during data acquisition or quantification.

## Conflict of Interest

The authors declare no conflict of interest.

## Author Contributions

L.L. and L.S. contributed equally to this work. H.K. and J.Z. conceived and designed the research and analysis; L.L., J.Z., and L.S. contributed to the data collection; L.L., W.Y., and H.K. performed the data analysis and wrote the paper with assistance from all authors. All authors contributed to the analysis of the results and the discussion in the manuscript.

## Supporting information



Supporting Information

## Data Availability

All data needed to evaluate the conclusions are present in the paper. Data are also available from the corresponding author upon request.
