# Supplementary material for: Sparse Nanocrystals Enable Ultra‐Low Coercivity and Remarkable Mechanical Robustness in High‐Entropy Amorphous Alloy
Source: Adv Sci (Weinh). 2025 Jul 12;12(38):e03546. doi: 10.1002/advs.202503546 (PMC12520540; doi:10.1002/advs.202503546)
Supplement: Supplementary file 1 — Supporting Information [file ADVS-12-e03546-s001.docx]

**Supporting Information**

**Sparse nanocrystals enable ultra-low coercivity and remarkable mechanical robustness in high-entropy amorphous alloy**

Lichen Liu^a,b,#^, Liliang Shao^b,c,#^, Yan Ma^a^, Zhilin Wen^b,d^, Jing Zhou^b,*^, Yuqiang Yan^b^, Haibo Ke^b,*^, Weiming Yang^a,*^, Weihua Wang^b,c^

^#^These authors contributed equally to this work.

^*^Correspondent: Jing Zhou ([zhoujing@sslab.org.cn](mailto:zhoujing@sslab.org.cn)), Weiming Yang ([wmyang@cumt.edu.cn](mailto:wmyang@cumt.edu.cn)), Haibo Ke ([kehaibo@sslab.org.cn](mailto:kehaibo@sslab.org.cn))

This PDF file includes:

Supplementary Figures S1-9 and Table S1


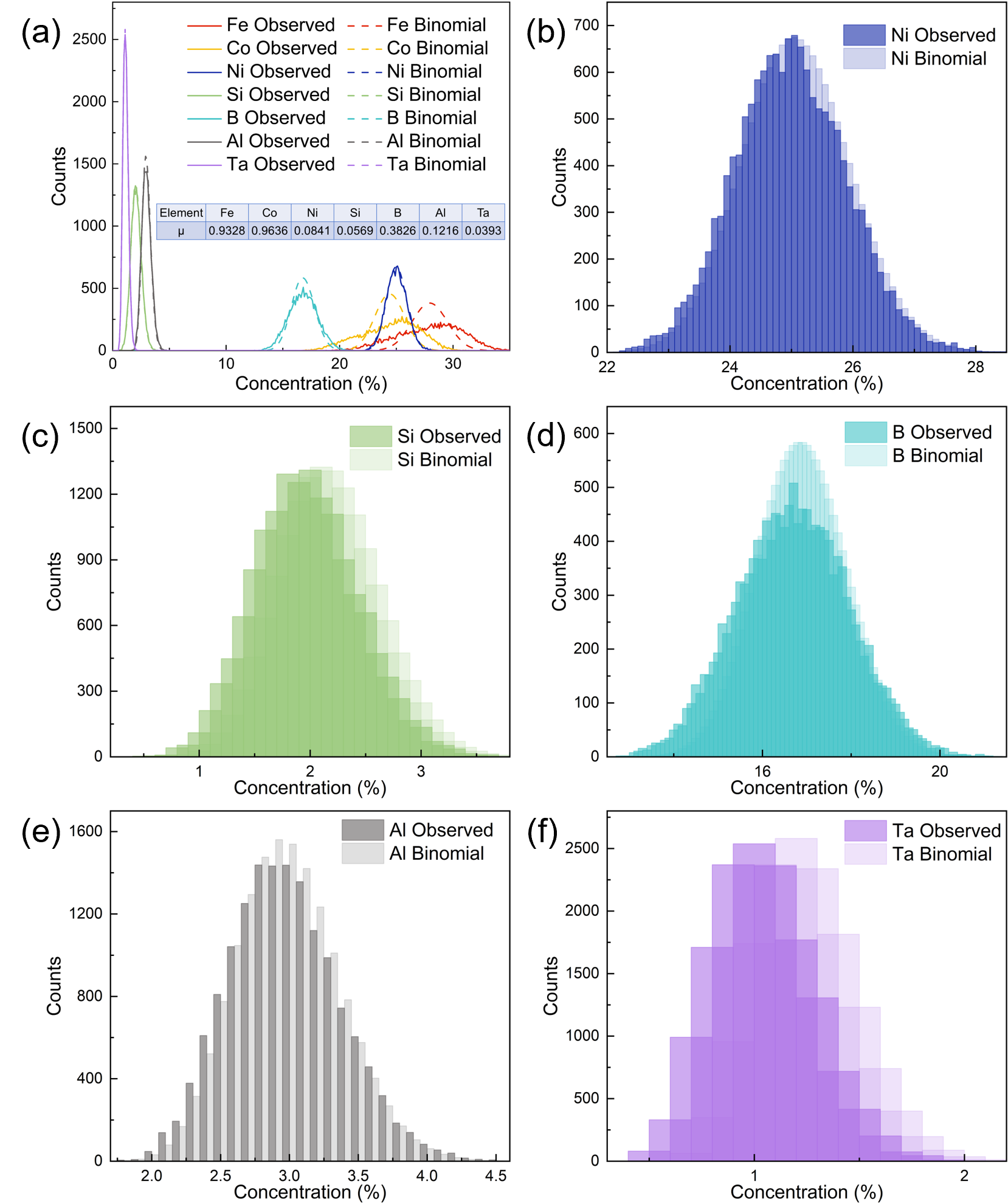


**Figure S1.** Binomial distribution of experimental data in comparison to the theoretical binomial random distribution.


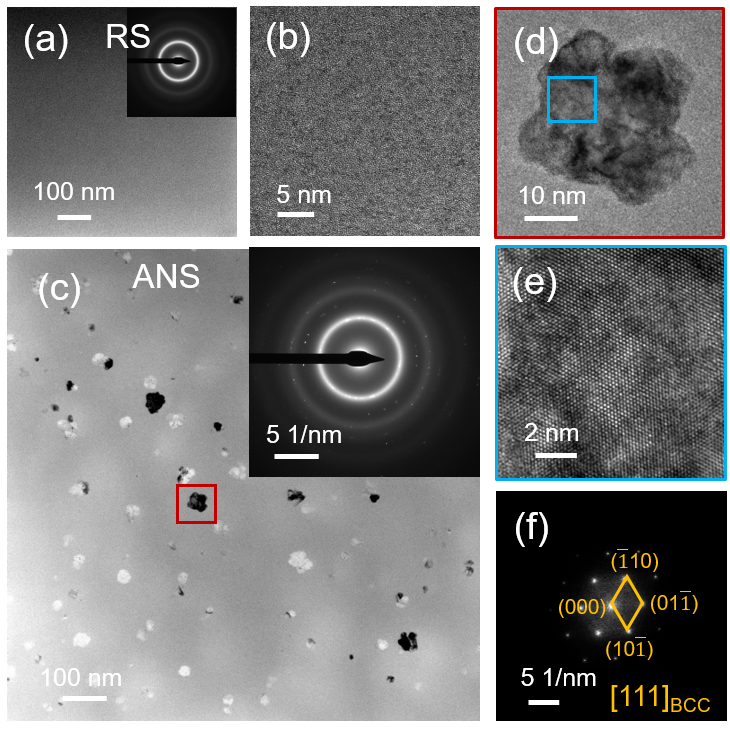


**Figure S2.** Microscopic structures in the RS and ANS states: (a) TEM image of annealed sample with 30 min of heat treatment at 673 K. The inset shows an SAED pattern. (b) HRTEM images of the fully amorphous region in (a). (c) TEM image of annealed sample with 4 min of heat treatment at 743 K. The inset shows an SAED pattern. (d) Enlarged image of the red square area in (c). (e) Enlarged image of the blue square area in (d). (f) FFT pattern of (e).


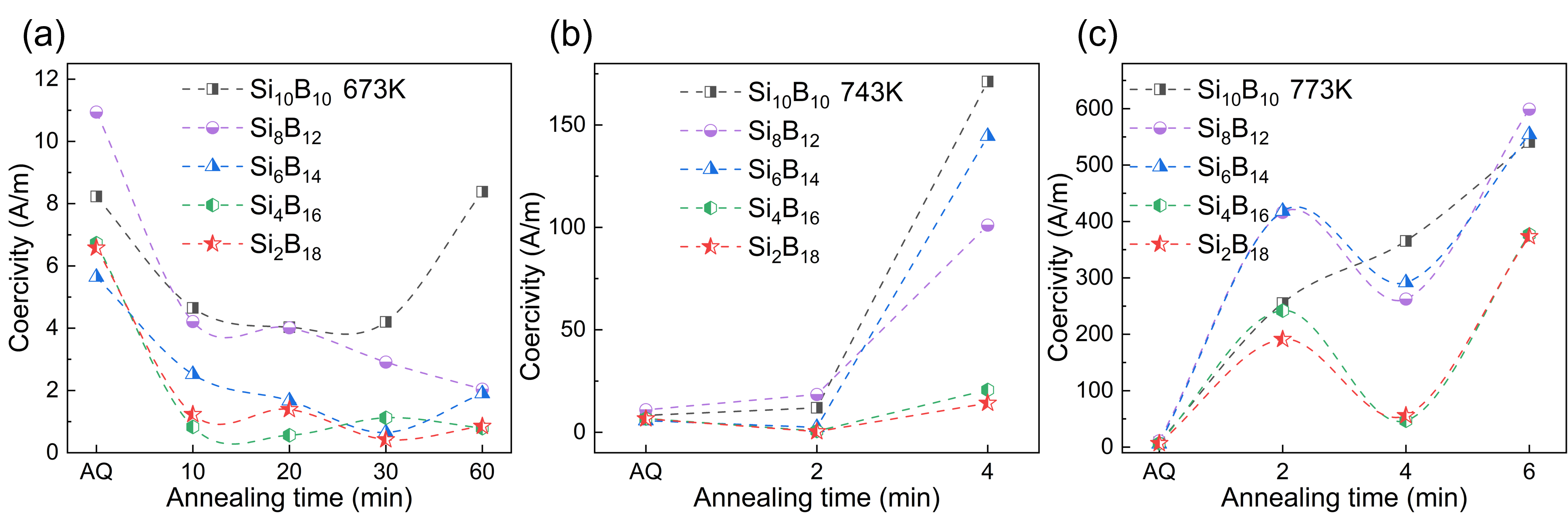


**Figure S3.** The coercivity of different heat treatment temperature and time of various materials.


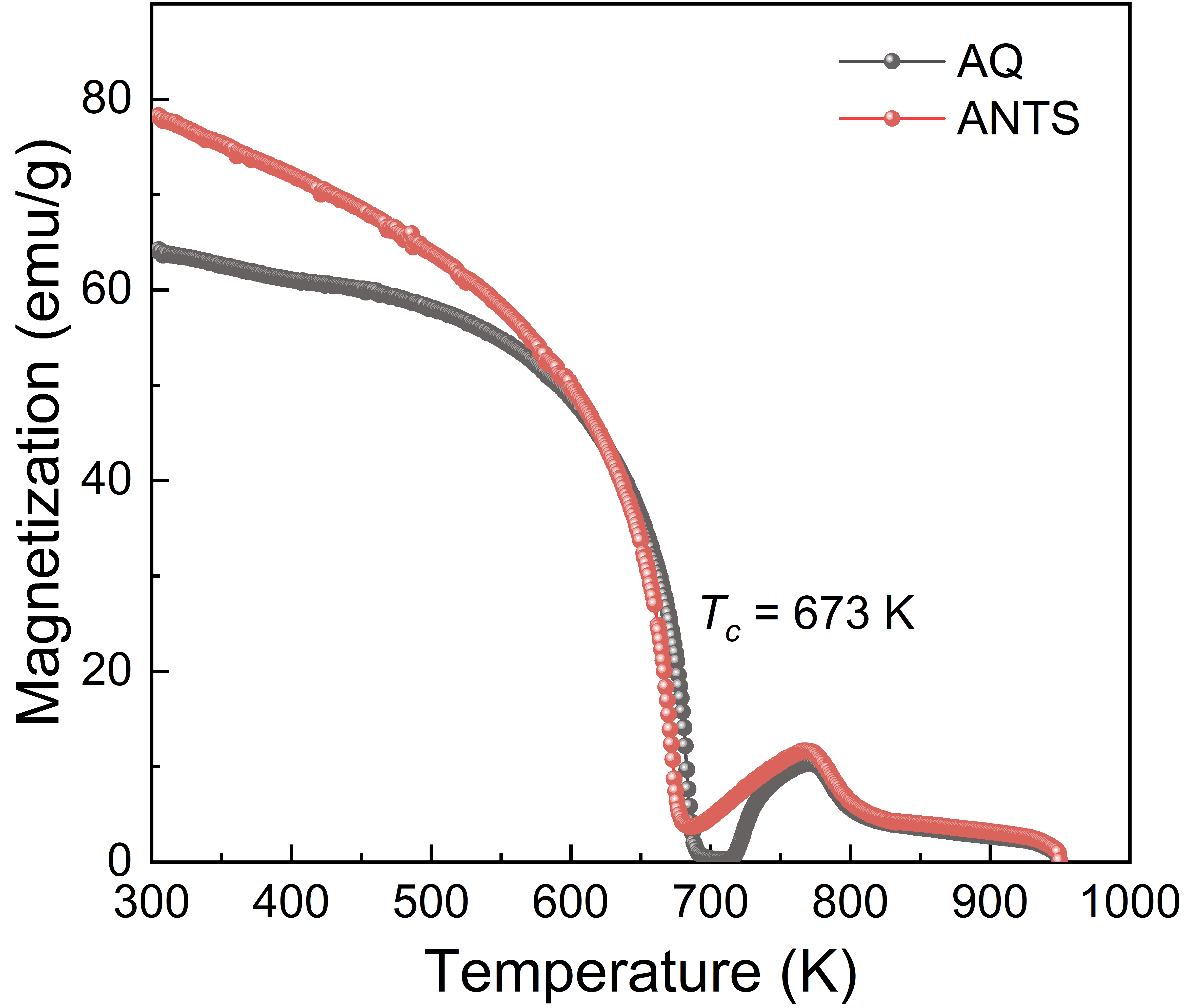


**Figure S4.** The *M-T* curves measured in the temperature range 300-950 K under a magnetic field of 200 Oe of AQ and ANTS ribbons.


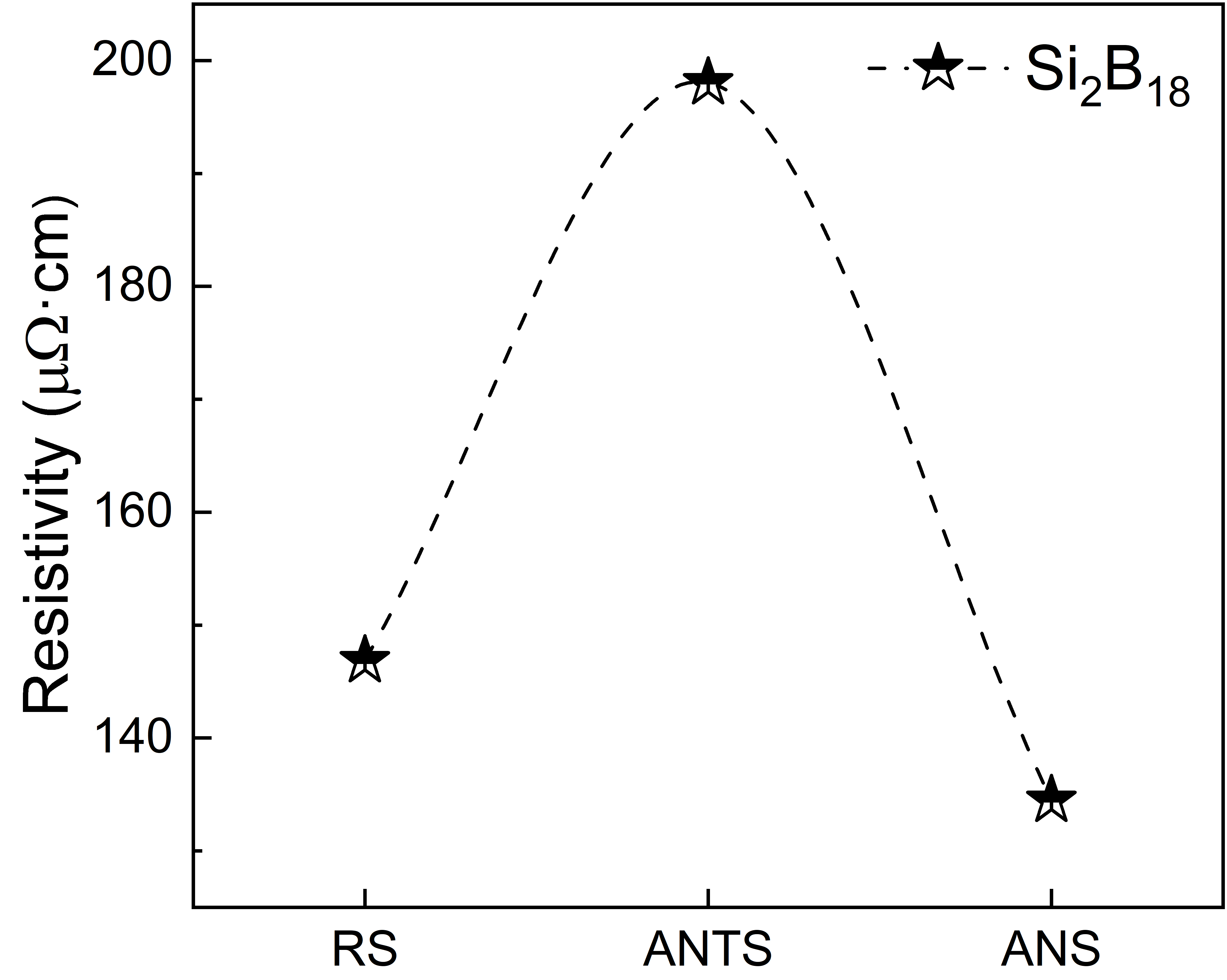


**Figure S5.** The resistivity of the various states.


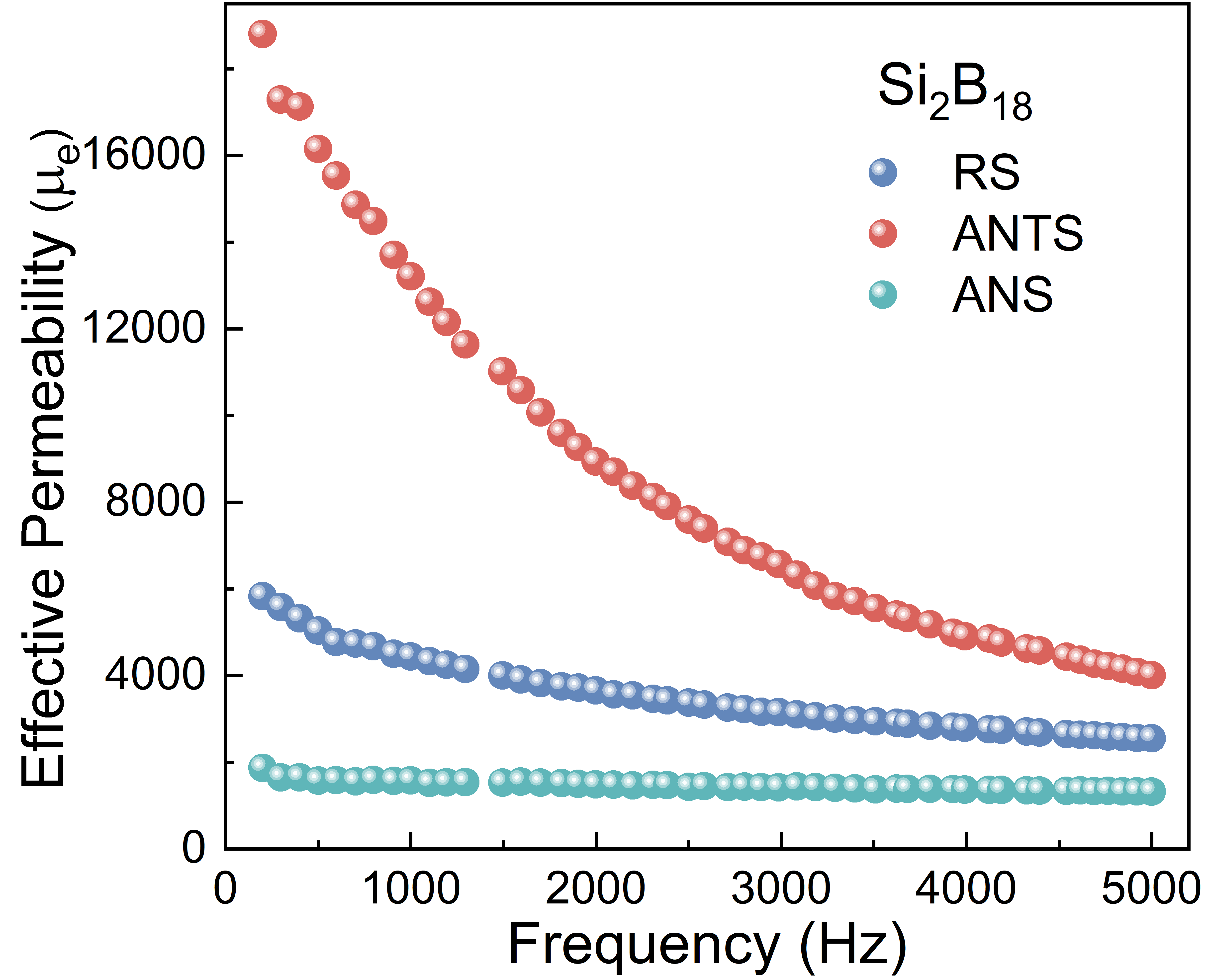


**Figure S6.** The effective permeability of the various states.


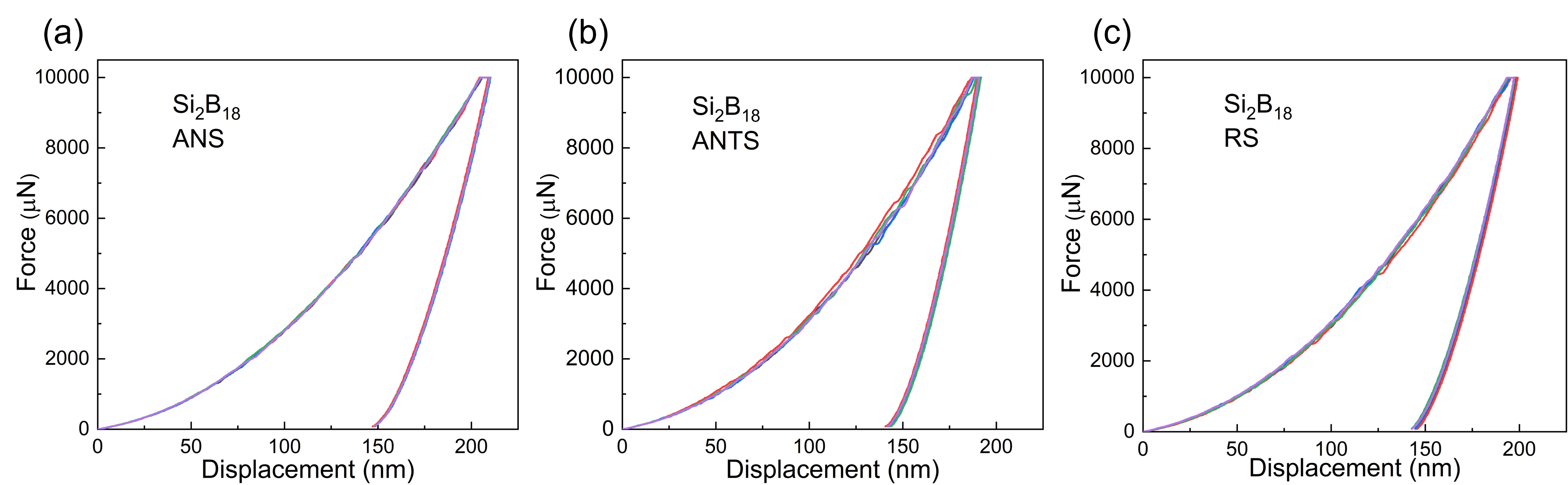


**Figure S7.** The nanoindentation curves obtained from multiple experiments of the various states.


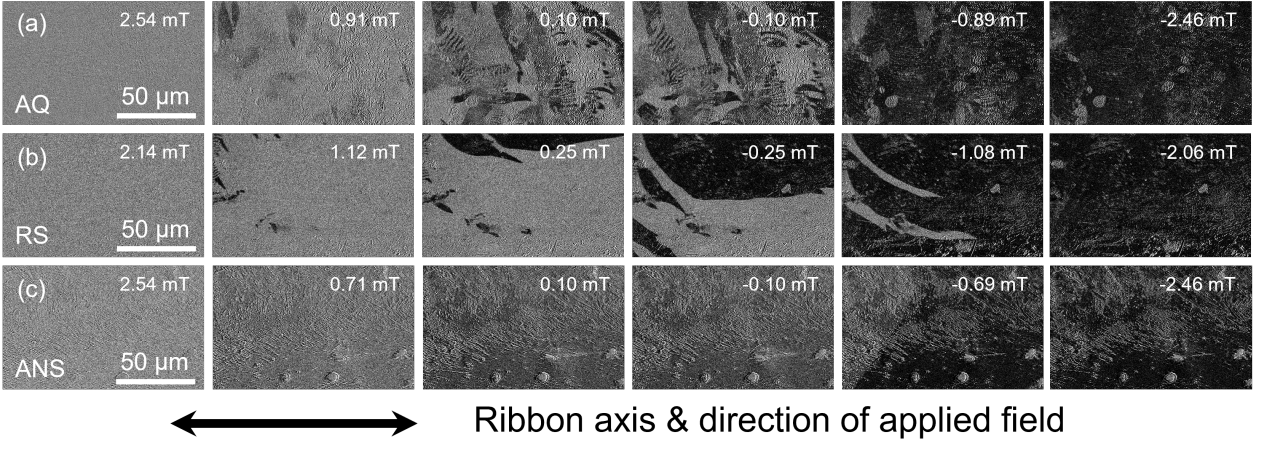


**Figure S8.** Magneto-optical Kerr subset images showing evolution of the magnetic domain structure.


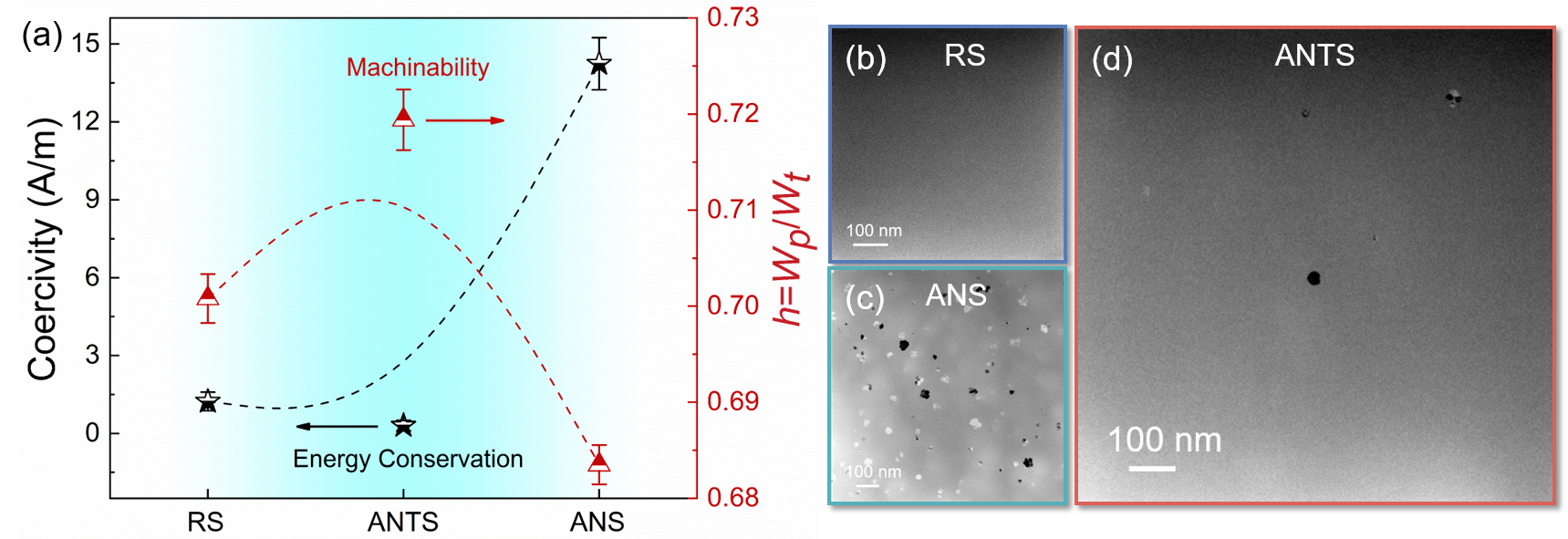


**Figure S9.** Balance of the mechanical-magnetic properties under order modulation.

**Table S1.** The saturation magnetic flux density (*B*_s_), coercivity (*H*_c_), resistivity (*ρ*), curie temperature (*T*_c_) and effective magnetic permeability (*µ_e_*) of the various soft-magnetic materials, including traditional crystalline alloys, Fe-based amorphous alloys, Co-based amorphous alloys, nanocrystalline alloys, high entropy alloys and the Fe_26_Co_25_Ni_25_Al_3_Ta_1_Si_2_B_18_ alloy in this paper.

| **Materials** | ***B*_s_**  **(T)** | ***H*_c_**  **(A/m)** | ***ρ***  **(μΩ·cm)** | ***T*_c_**  **(K)** | ***µ_e_***  **(at 1 kHz)** |
| --- | --- | --- | --- | --- | --- |
| Fe [1] | 2.16 | 18.4 | 9.7 | — | — |
| Fe-3 wt.%Si [2] | 2.00 | 46 | 51 | — | ~2000 |
| Fe_100-x_B_x_ [2] | 1.89-1.95 | 6.4-13.6 | 49-65 | 500-550 | — |
| Fe_85_B_13_Ni_2_ [2] | 1.90 | 3.8 | 62 | 500-550 | — |
| Fe_32.6_Co_27.7_Ni_27.7_Ta_5_Al_7_ [3] | 0.95 | 78 | 103 | 668-705 | — |
| Fe_20_Co_20_Ni_20_Al_20_Cr_20_ [4] | 0.15-0.8 | 470-1320 | 164-187 | ~1080 | — |
| FeCoNiMn_0.25_Al_0.25_ [5] | 0.91 | 230-625 | 91-110 | — | 72-480 |
| Fe_78_Si_9_B_13_ [6] | 1.56 | 2.4 | 137 | — | 10000 |
| Fe_90_Zr_7_B_3_ [7] | 1.63 | 5.6 | 44 | — | 22000 |
| Fe_80-x-y_Nb_5_B_14_Cu_1_Co_x_Ni_y_ | 0.84-0.91 | 3.2-188.6 | 121-163 | — | — |
| Fe_84_B_8.5_Si_4.5_P_3_ [8] | 1.7 | 6.2 | — | 581 | 8300 |
| Fe_84_B_8.5_Si_4.5_P_3_ [8] | 1.65 | 17.4 | — | 565 | 7300 |
| (Fe_1-x_Co_x_)_88_Zr_7_B_4_Cu_1_ [9] | 1.6-2.1 | 10 | — | ~1073 | 1800 |
| Fe_80_(Nb_1-x_Mo_x_)_5_B_15_ [10] | 0.84-1.04 | 11.9-26.3 | 116-150 | — | — |
| Co_63_Fe_7_Zr_6_Ta_4_B_20_ [11] | 0.54 | 3.4 | 193 | — | — |
| (CoFeMo)_73_(SiB)_27_ [12] | 0.55 | — | 135 | 483 | — |
| (CoFeMnMo)_77_(SiB)_23_ [12] | 0.8 | — | 130 | 623 | — |
| (CoFeMn)_80_(SiB)_20_ [12] | 1.0 | — | 115 | 773 | — |
| Fe_26_Co_25_Ni_25_Al_3_Ta_1_Si_2_B_18_ | 1.0 | 0.3 | 198 | 673 | 13200 |

**References**

[1] W. Zhang, R. Li, J. Wang, T. Zhang, Y. Gao, T. Zhang, *Mater. Design* **2024**, 246, 113311.

[2] R. Parsons, B. Zang, K. Onodera, H. Kishimoto, T. Shoji, A. Kato, K. Suzuki, *J. Magn. Magn. Mater.* **2019**, 476, 142.

[3]L. Han, F. Maccari, I. R. Souza Filho, N. J. Peter, Y. Wei, B. Gault, O. Gutfleisch, Z. Li, D. Raabe, *Nature* **2022**, 608, 310.

[4] C. Zhao, J. Li, Y. He, J. Wang, W.Y. Wang, H. Kou, J. Wang, *J. Alloys Compd.* **2020**, 820, 153407.

[5] P. Li, A. Wang, C.T. Liu, *J. Alloys Compd.* **2017**, 694, 55.

[6] L.T. Kabacoff, *J. Appl. Phys.* **1982**, 53, 8098.

[7] K. Suzuki, A. Makino, A. Inoue, T. Masumoto, *J. Appl. Phys.* **1991**, 70, 6232.

[8] F.L. Kong, C.T. Chang, A. Inoue, E. Shalaan, F. Al-Marzouki, *J. Alloys Compd.* **2014**, 615, 163.

[9] M.A. Willard, D.E. Laughlin, M.E. McHenry, D. Thoma, K. Sickafus, J.O. Cross, *J. Appl. Phys.* **1998**, 84, 6773.

[10] M. Zhu, M. Zhang, L. Yao, R. Nan, Z. Jian, F.e. Chang, *Vacuum* **2019**, 163, 368.

[11] K.V. Rao, *Butterworth-Heinemann* **1983**, 401.

[12] G. Herzer, H.R. Hilzinger, *Phys. Scripta* **1988**, 34, 22.
